# Supplementary material for: Population decline in a ground-nesting solitary squash bee (Eucera pruinosa) following exposure to a neonicotinoid insecticide treated crop (Cucurbita pepo)
Source: Sci Rep. 2021 Feb 19;11:4241. doi: 10.1038/s41598-021-83341-7 (PMC7896084; doi:10.1038/s41598-021-83341-7)
Supplement: Supplementary file 1 — Supplementary Information [file 41598_2021_83341_MOESM1_ESM.docx]

Population decline in a ground-nesting solitary squash bee (*Eucera pruinosa*) following exposure to a neonicotinoid insecticide treated crop (*Cucurbita pepo*)

D. Susan Willis Chan, Nigel E. Raine

School of Environmental Sciences, University of Guelph, Guelph, Ontario, N1G 2W1, Canada

Correspondence: [dchan05@uoguelph.ca](about:blank); nraine@uoguelph.ca

**Supplementary Information**

Table S1. Model comparisons including descriptions and associated Akaike Information Criteria (AIC) for each model. Treatment effects refer to systemic insecticide treatment applied (Admire, FarMore F1400 or Coragen) or an untreated control. Flower effects refer to the number of flowers per hoop house in which the treatment was applied. Where data were collected in both 2017 and 2018, then year was included as a fixed effect in the model. Models for each dependent variable with lowest AIC values (shown in bold) were used for statistical analyses.

| **Dependent Variable** | **Model Iteration** | **Model Description** | **AIC** |
| --- | --- | --- | --- |
| Total Nests Initiated | 1 | Total Nests = Treatment + Year + Treatment*Year + Flowers, Random effect of Block | 104.3 |
|  | **2** | **Total Nests = Treatment + Year + Treatment*Year, Random effect of Block** | **101.8** |
|  | 3 | Total Nests = Treatment + Year + Flowers, Random effect of Block | 119.5 |
|  | 4 | Total Nests = Treatment + Year, Random effect of Block | 117.4 |
| Unharvested Pollen | **1** | **Unharvested Pollen = Treatment, Repeated effect of Observation Day, Random effect of Block** | **2307** |
| Total Offspring | **1** | **Total Offspring = Treatment + Year + Treatment*Year + Flowers, Random effect of Block** | **132.5** |
|  | 2 | Total Offspring = Treatment + Year + Treatment*Year, Random effect of Block | 163.5 |
|  | 3 | Total Offspring = Treatment + Year + Flowers, Random effect of Block | 163.5 |
|  | 4 | Total Offspring = Treatment + Year, Random effect of Block | 167.7 |
| Sex Ratio | 1 | Sex Ratio = Treatment + Year + Treatment*Year +Flowers, Random effect of block | 80.9 |
|  | **2** | **Sex Ratio = Treatment + Year** | 76.7 |
| % Fruit Set | **1** | **% Fruit Set = Treatment** | **3.2** |
| % Marketable Fruit | **1** | **% Marketable Fruit=Treatment + Year + Treatment*Year, Random effect of Block** | **-14.1** |

Table S2. Test statistics for each fixed effect associated with the best fitting model (Table S1) for each dependent variable (total nests initiated, total offspring, sex ratio, unharvested pollen, percentage fruit set, and percentage marketable fruit). If an effect was not included in the best fitting model this is indicated by “n/a”.

| **Model** | **Fixed Effects** | | | |
| --- | --- | --- | --- | --- |
|  | **Treatment** | **Year** | **Treatment*Year** | **Number of Flowers** |
| **Total Nests Initiated**  = Treatment + Year + Treatment*Year,  Random effect of Block | F_(3,14)_ = 7.33  p = 0.003 | F_(1,14)_ = 6.75  p = 0.021 | F_(3,14)_ = 0.67  p = 0.583 | n/a |
| **Unharvested Pollen** = Treatment, Repeated effect of Observation Day, Random effect of Block | F_(3,114)_ = 37.82  p < 0.001 | n/a | n/a | n/a |
| **Total Offspring** = Treatment + Year + Treatment*Year + Flowers,  Random effect of Block | F_(3,13)_ = 14.19  p = 0.0002 | F_(1,13)_ = 11.79  p = 0.004 | F_(3,13)_ = 3.81  p = 0.037 | F_(1,13)_ = 10.44  p = 0.007 |
| **Sex Ratio** = Treatment + Year | F(_3,14)_ = 0.08  p = 0.968 | F_(1,14)_ = 6.73  p = 0.021 | n/a | n/a |
| **% Fruit Set** = Treatment | F_(3,8)_ = 0.16  p = 0.919 | n/a | n/a | n/a |
| **% Marketable Fruit** = Treatment + Year + Treatment*Year, Random effect of Block | F_(3,14)_ = 0.14  p = 0.936 | F_(1,14)_ = 9.22  p = 0.009 | F_(3,14)_ = 1.17  p = 0.355 | n/a |

Table S3. Post-hoc pairwise comparisons between treatments (Admire, or Coragen, or FarMore F1400) and untreated control for dependent variables (total nests, total offspring, unharvested pollen) showing significant effects in best fit models (see Table S2). Tukey-Kramer adjustments were applied to p-values to account for multiple pairwise comparisons. Identical t-values for total nest comparisons reflect identical mean values of control, Coragen, and FarMore F1400 treatments over both years (Table 1). Lower and upper 95% confidence intervals are provided.

| **Dependent Variable** | **Treatments Compared** | **DF** | **t-Value** | **p > \|t\|** | **Adj p** |
| --- | --- | --- | --- | --- | --- |
| **Total Nests** | Admire vs Control | 13 | -3.83 | 0.002 | 0.009 |
|  | Admire vs Coragen | 13 | -3.66 | 0.003 | 0.009 |
|  | Admire vs FarMore F1400 | 13 | -3.68 | 0.003 | 0.009 |
|  | Control vs Coragen | 13 | -0.00 | 0.988 | > 0.999 |
|  | Control vs FarMore F1400 | 13 | -0.00 | 0.998 | > 0.999 |
|  | Coragen vs FarMore F1400 | 13 | -0.00 | 0.999 | > 0.999 |
| **Total Offspring** | Admire vs Control | 15 | -5.98 | <0.001 | 0.0002 |
|  | Admire vs Coragen | 15 | -5.33 | 0.0001 | 0.001 |
|  | Admire vs FarMore F1400 | 15 | -3.55 | 0.004 | 0.016 |
|  | Control vs Coragen | 15 | 0.80 | 0.439 | 0.854 |
|  | Control vs FarMore F1400 | 15 | 2.59 | 0.022 | 0.091 |
|  | Coragen vs FarMore F1400 | 15 | 1.82 | 0.092 | 0.309 |
| **Unharvested Pollen** | Admire vs Control | 114 | 9.38 | <0.001 | <0.001 |
|  | Admire vs Coragen | 114 | 6.72 | <0.001 | <0.001 |
|  | Admire vs FarMore F1400 | 114 | 9.02 | <0.001 | <0.001 |
|  | Control vs Coragen | 114 | -2.65 | 0.009 | 0.044 |
|  | Control vs FarMore F1400 | 114 | -0.36 | 0.722 | 0.984 |
|  | Coragen vs FarMore F1400 | 114 | 2.30 | 0.023 | 0.105 |

**Table S4.** Raw data for flower count, total females, total nests, total offspring, male offspring, and female offspring and calculated indices (percentage of male, and sex ratio of, offspring produced) for each pesticide treatment applied to a squash crop in twelve net-covered hoop houses occupied by a captive population of hoary squash bees (*Eucera* (*Peponapis*) *pruinosa*).

| **Treatment** | **Hoop House** | **Year** | **Flower Count** | **Number of Female Bees** | **Total Nests** | **Total Offspring** | **Male Offspring** | **Female Offspring** | **Percent Males (%)** | **Sex Ratio** |
| --- | --- | --- | --- | --- | --- | --- | --- | --- | --- | --- |
| Admire | A1 | 2017 | 41.2 | 8 | 0 | 3 | 1 | 2 | 33 | 0.5 |
| Admire | B4 | 2017 | 52.2 | 8 | 0 | 1 | 1 | 0 | 100 | - |
| Admire | C3 | 2017 | 43.0 | 8 | 1 | 6 | 2 | 4 | 33 | 0.5 |
| Admire | A1 | 2018 | 56.0 | 2 | 0 | 0 | 0 | 0 | - | n/a |
| Admire | B4 | 2018 | 54.3 | 0 | 0 | 0 | 0 | 0 | - | n/a |
| Admire | C3 | 2018 | 53.0 | 4 | 8 | 10 | 8 | 2 | 80 | 4.0 |
| Control | A2 | 2017 | 27.8 | 8 | 4 | 5 | 3 | 2 | 60 | 1.5 |
| Control | B1 | 2017 | 44.5 | 8 | 13 | 22 | 13 | 9 | 59 | 1.4 |
| Control | C4 | 2017 | 56.2 | 8 | 9 | 42 | 15 | 27 | 36 | 0.6 |
| Control | A2 | 2018 | 43.6 | 2 | 6 | 31 | 19 | 12 | 61 | 1.6 |
| Control | B1 | 2018 | 54.1 | 9 | 8 | 58 | 44 | 14 | 76 | 3.1 |
| Control | C4 | 2018 | 41.6 | 27 | 19 | 37 | 25 | 12 | 68 | 2.1 |
| Coragen | A3 | 2017 | 45.8 | 8 | 7 | 24 | 5 | 19 | 21 | 0.3 |
| Coragen | B2 | 2017 | 41.0 | 8 | 8 | 13 | 6 | 7 | 46 | 0.9 |
| Coragen | C1 | 2017 | 57.3 | 8 | 9 | 17 | 5 | 12 | 29 | 0.4 |
| Coragen | A3 | 2018 | 32.4 | 12 | 14 | 17 | 7 | 10 | 41 | 0.7 |
| Coragen | B2 | 2018 | 46.3 | 7 | 10 | 49 | 45 | 4 | 92 | 11.3 |
| Coragen | C1 | 2018 | 57.6 | 12 | 11 | 62 | 35 | 27 | 56 | 1.3 |
| FarMore FI400 | A4 | 2017 | 51.2 | 8 | 2 | 18 | 8 | 10 | 44 | 0.8 |
| FarMore FI400 | B3 | 2017 | 49.0 | 8 | 8 | 17 | 10 | 7 | 59 | 1.4 |
| FarMore FI400 | C2 | 2017 | 63.7 | 8 | 8 | 30 | 9 | 21 | 30 | 0.4 |
| FarMore FI400 | A4 | 2018 | 44.9 | 10 | 18 | 11 | 8 | 3 | 73 | 2.7 |
| FarMore FI400 | B3 | 2018 | 34.6 | 6 | 10 | 15 | 9 | 6 | 60 | 1.5 |
| FarMore FI400 | C2 | 2018 | 41.1 | 21 | 13 | 34 | 30 | 4 | 88 | 7.5 |

Table S5. Sampling regime for each study including the year of observation, the number of observation days and the observation dates for the nest establishment, flower counts, and pollen harvest studies undertaken.

| **Study** | **Description** | **Year** | **Observation** | |
| --- | --- | --- | --- | --- |
|  |  |  | **Days** | **Dates** |
| Nest Establishment | Observers searched for active nests within each hoop house at the start of each observation day and marked each nest with a numbered and dated marker (Extended Data Fig.6) | 2017 | 10 | August 4,7,10,11,15,17,18,21,23,24 |
|  |  | 2018 | 8 | August 7,9,10,13,14,15,20,23 |
| Flower Counts | The total number of staminate and pistillate flowers in each hoop house was counted during the daily flowering period | 2017 | 6 | August 7,11,15,18,21,23 |
|  |  | 2018 | 8 | August 7,9,10,13,14,15,20,23 |
| Pollen Harvest | Pollen harvest by the whole population of hoary squash bees within a hoop house was evaluated by measuring the amount of unharvested pollen remaining on anthers of staminate flowers in each hoop house at the end of the daily foraging period | 2017 | 2 | August 17, 23 |
| Fruit set | Female flowers were marked each day for 10 days during the squash crop flowering period. At the end of the season, the number of flowers that had set fruit were counted and related to the total number of flowers | 2017 | 10 | August 4,7,10,11,15,17,18,21,23,24 |

**Table S6.** Mean concentration (ppb) of residues in soil from hoop houses treated with Admire (applied to soil at planting) or FarMore FI400 (applied as a seed coating) or Coragen (spray applied to foliage at the 5-leaf stage) or an untreated control. All treatments were applied to squash (2017: Table Star; 2018: Celebration) in net-covered hoop houses before hoary squash bees were active. The mean concentration of residues in cells marked with a dash were below the minimum quantifiable or detectable limits. Six samples were taken in each treatment (i.e. 2 samples on each of 3 days) during the bee-active period in 2017 (July 17, August 4, August 18) and 2018 (July 18, August 15, August 23).

|  | **Hoop** | **Mean Residue Concentration Detected (ppb)** | | | | | | | |
| --- | --- | --- | --- | --- | --- | --- | --- | --- | --- |
|  | **House** | **imidacloprid** | | **clothianidin** | | **thiamethoxam** | | **chlorantraniliprole** | |
| **Treatment** | **ID** | **2017** | **2018** | **2017** | **2018** | **2017** | **2018** | **2017** | **2018** |
| Admire | A1 | 25.6 | 142.6 | 0.1 | - | - | - | - | - |
| Control | A2 | - | - | 0.2 | - | - | - | - | - |
| Coragen | A3 | - | - | 1.0 | 1.3 | - | - | 0.6 | 11.3 |
| FarMore F1400 | A4 | - | - | 1.2 | 1.2 | 0.1 | 1.9 | - | - |
| Control | B1 | - | - | 3.6 | - | 9.1 | - | - | - |
| Coragen | B2 | - | - | 2.4 | - | - | - | - | 8.1 |
| FarMore F1400 | B3 | - | - | 3.1 | 3.6 | - | 16.6 | - | - |
| Admire | B4 | 11.3 | 44.6 | 1.7 | - | - | - | - | - |
| Coragen | C1 | - | - | 5.7 | - | - | - | - | 23.8 |
| FarMore F1400 | C2 | 31.5 | - | 2.6 | 1.6 | - | 1.1 | - | - |
| Admire | C3 | 48.0 | 88.8 | 3.5 | 1.0 | - | - | - | - |
| Control | C4 | - | - | 3.6 | 2.2 | - | - | - | - |


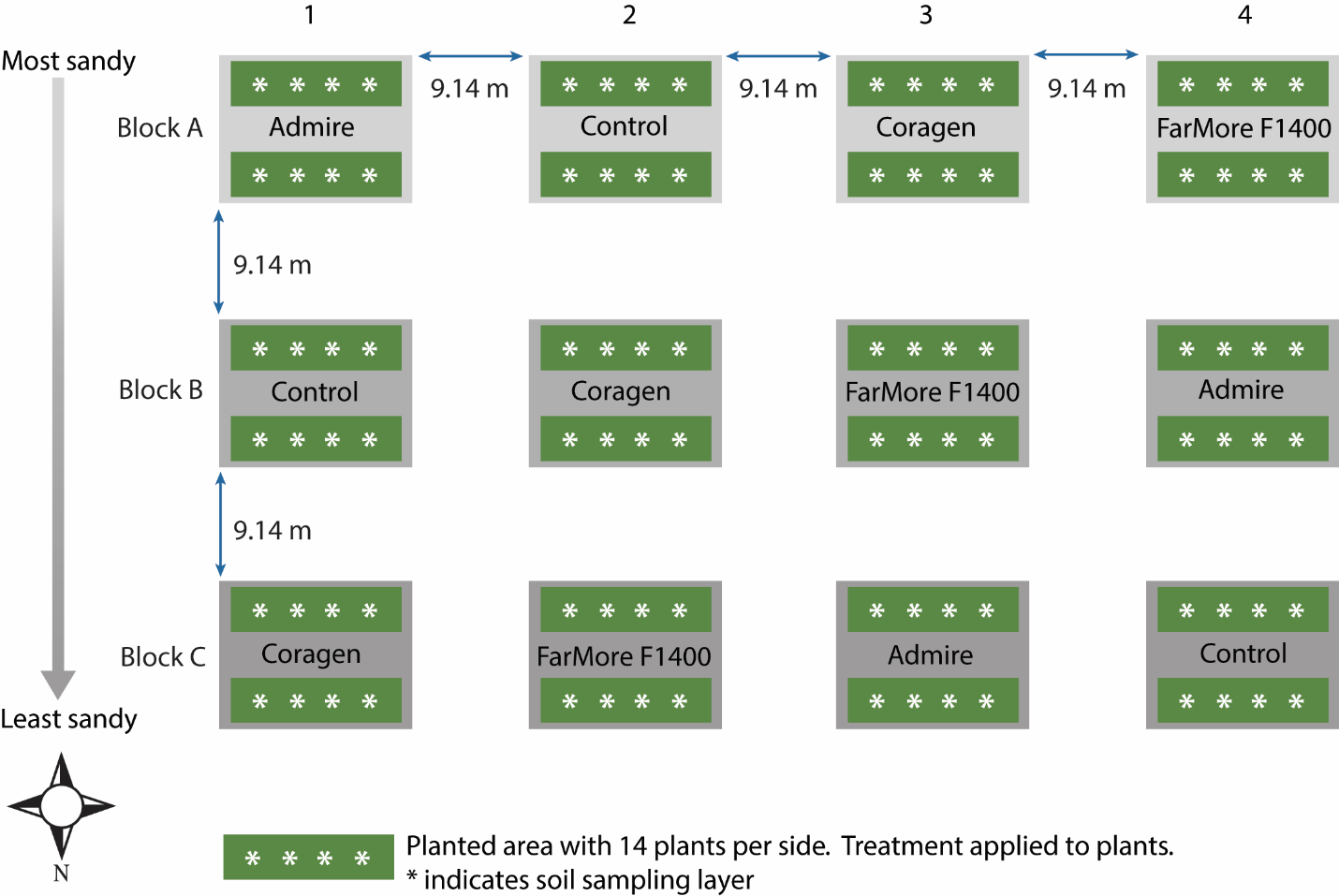


Figure S1. Layout of study site in Peterborough County, Ontario, showing the arrangement and spacing of 12 hoop houses (grey rectangles: A1-C4), the treatments applied to each one (Admire, Coragen or FarMore FI400, or untreated control), the two planted areas in each hoop house (green rectangles), the soil sampling layout (white asterisks), and the soil texture gradient from most to least sandy at the site.


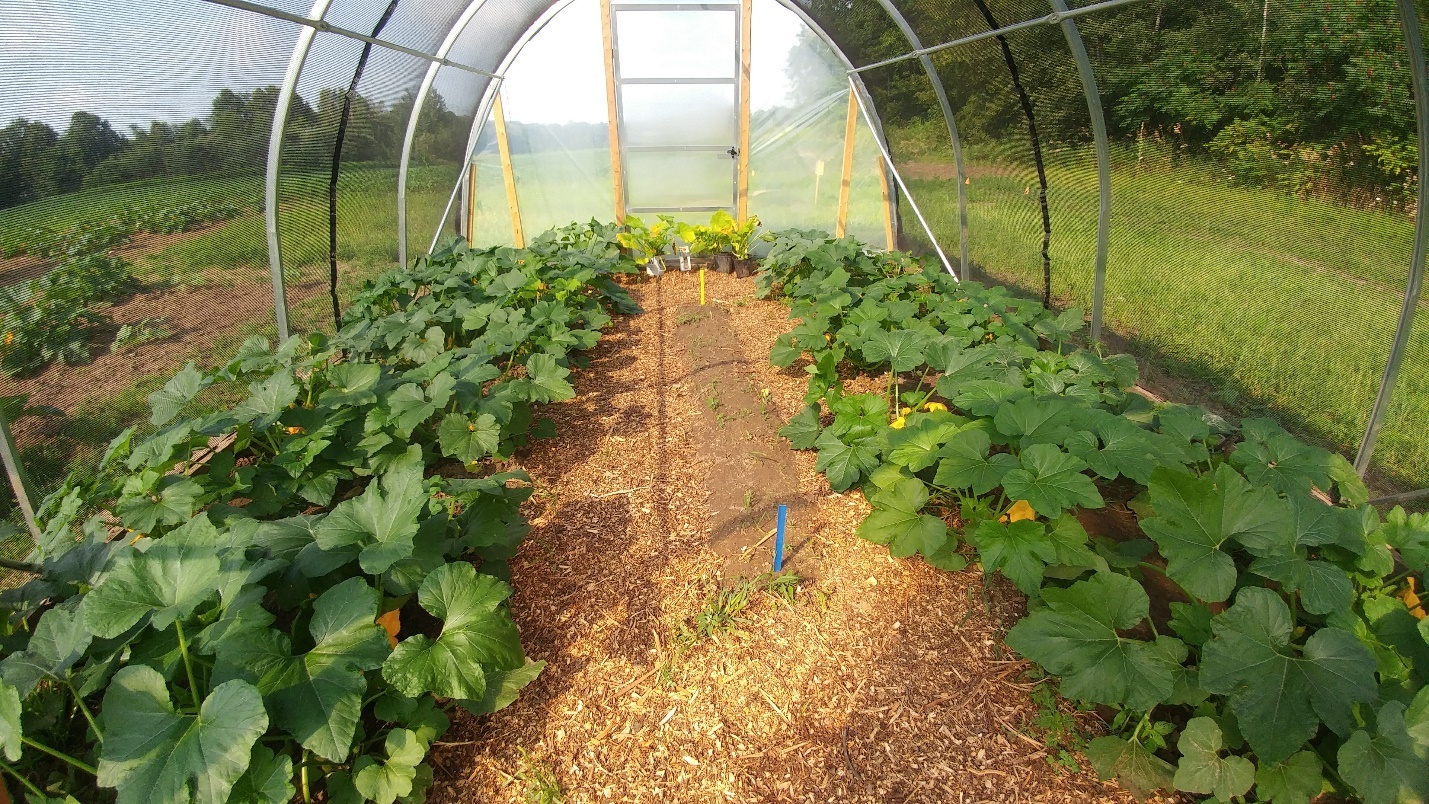


Figure S2. Hoop house at the study site in Peterborough County, Ontario, showing the areas planted with 28 acorn squash plants, the mulched pathways, and the bare nesting area (between the blue and yellow vertical markers). Hoop houses were covered with shade cloth allowing exterior conditions to prevail inside while preventing introduced hoary squash bees from escaping or other bees from entering. Hoary squash bees used both the mulched paths and the bare soil area to excavate nests. Photo: Beatrice Chan, used with permission.


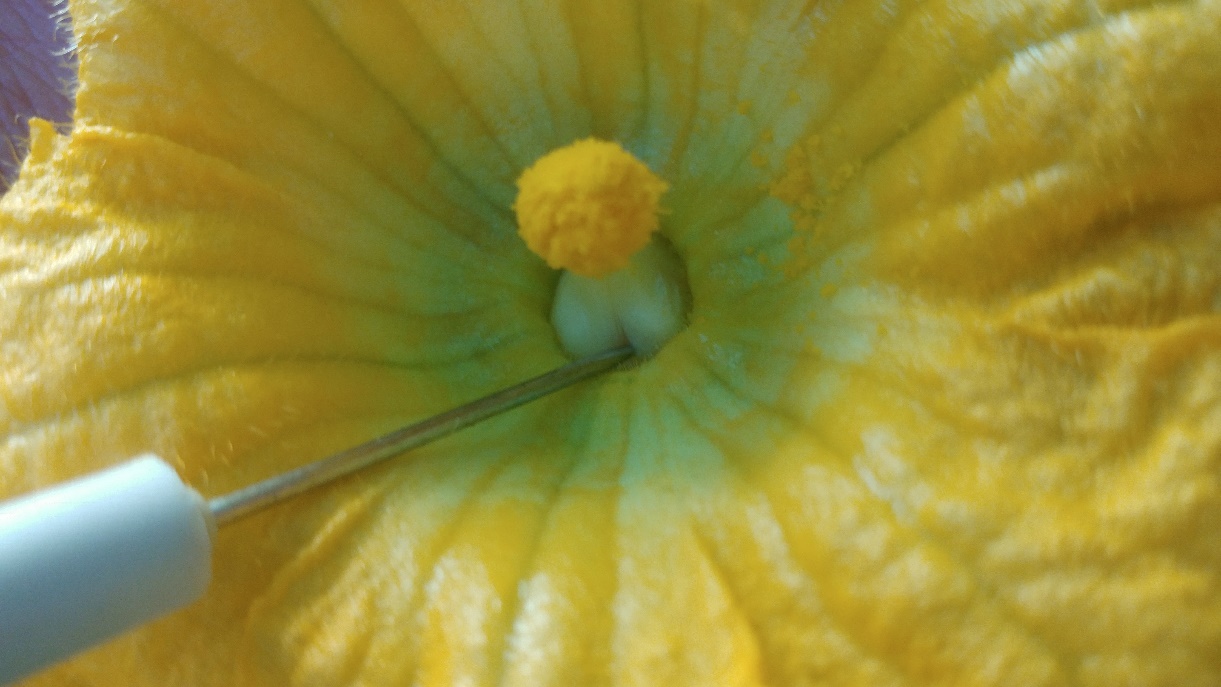


Figure S3. Staminate flower of Table Star variety of acorn squash. These flowers lacked access holes in the base of their fused stamen to allow bees to gather nectar from the enclosed nectaries. To solve this problem observers inserted a dissection needle into the base of the fused stamen to create three access holes for each staminate flower at the start of each day during the flowering period in 2017. Photo: Beatrice Chan, used with permission.


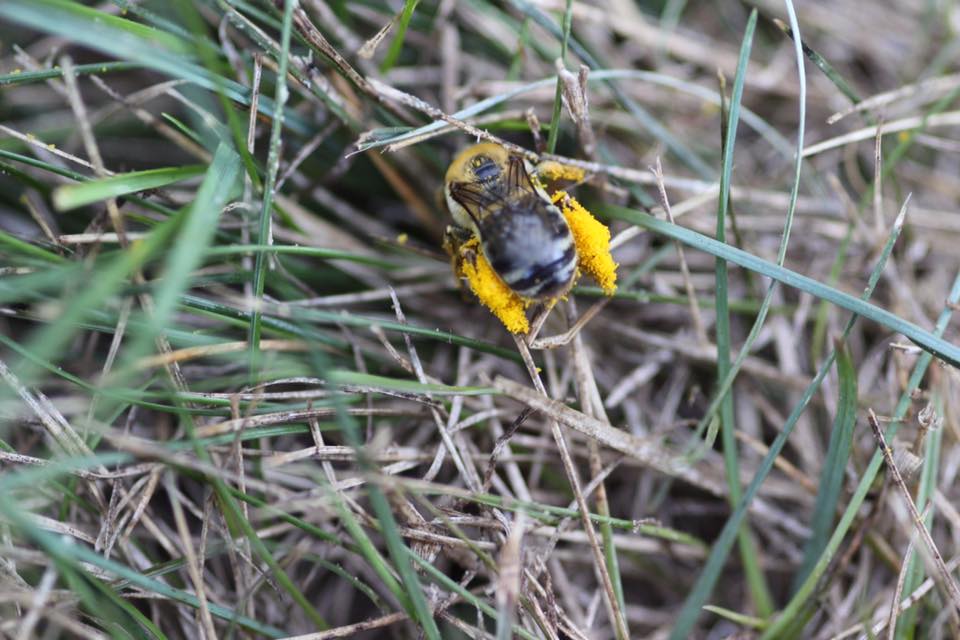


Figure S4. Hoary squash bee female (*Eucera* (*Peponapis*) *pruinosa*) preparing to enter her nest with a full load of yellow *Cucurbita* spp. pollen on her hind legs. Female bees, such as the one pictured here, were captured for the study as they entered their nests. Photo: Beatrice Chan, used with permission.


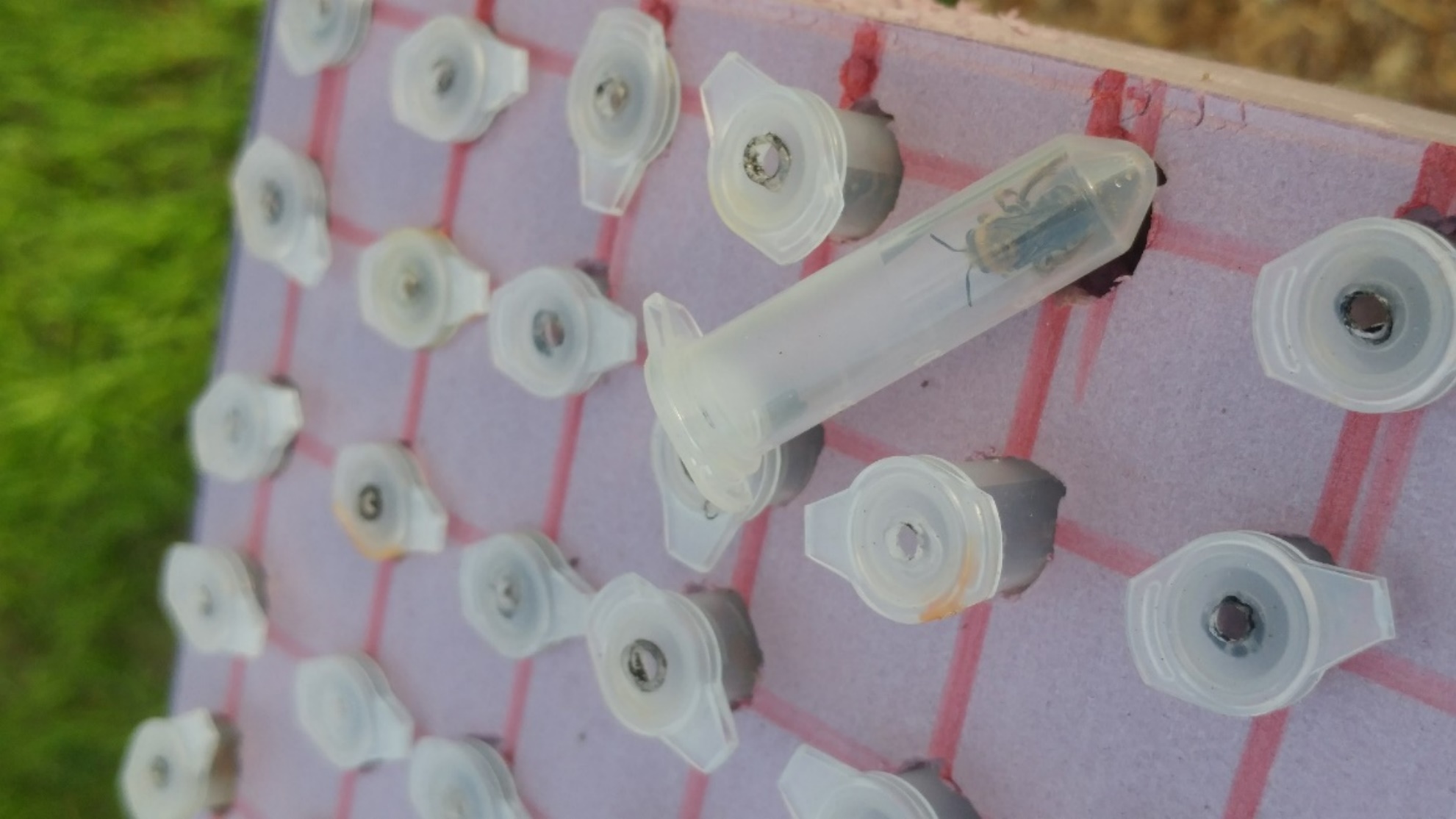


**Figure S5.** Aerated micro-centrifuge tubes used to contain and transport hoary squash bees (*Eucera* (*Peponapis*) *pruinosa*) from the site of capture in Guelph, Ontario to the study hoop houses in Peterborough County. Photo: Beatrice Chan, used with permission.

**
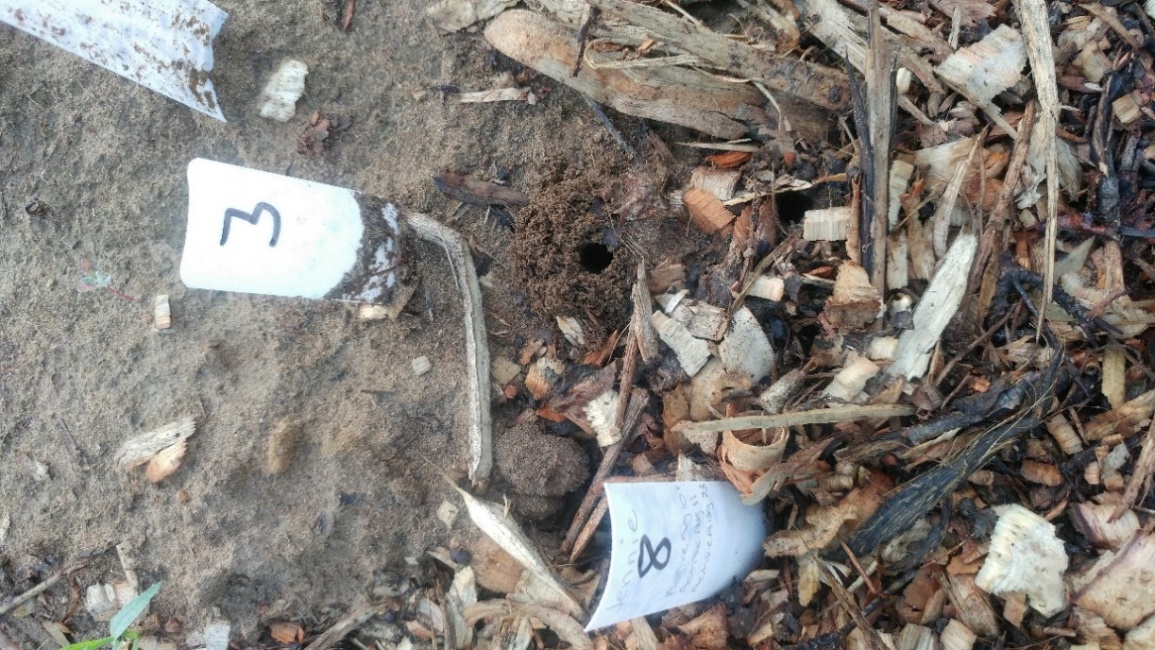
**

**Figure S6.** Aggregation of hoary squash bee nests within a study hoop house showing a nest entrance with tumulus of excavated soil (marked with a white arrow), and markers with identification numbers. These markers were used to enable observers to keep track of nests over the season and accurately record activity. Photo: Beatrice Chan, used with permission.
